# PU.1 Is Required for the Developmental Progression of Multipotent Progenitors to Common Lymphoid Progenitors

**DOI:** 10.3389/fimmu.2018.01264

**Published:** 2018-06-11

**Authors:** Swee Heng Milon Pang, Carolyn A. de Graaf, Douglas J. Hilton, Nicholas D. Huntington, Sebastian Carotta, Li Wu, Stephen L. Nutt

**Affiliations:** ^1^The Walter and Eliza Hall Institute of Medical Research, Parkville, VIC, Australia; ^2^Department of Medical Biology, University of Melbourne, Parkville, VIC, Australia; ^3^Oncology Research, Boehringer Ingelheim, Vienna, Austria; ^4^Institute for Immunology, Tsinghua University School of Medicine, Beijing, China

**Keywords:** PU.1, transcription factor, multipotent progenitor, common lymphoid progenitor, Rag1

## Abstract

The transcription factor PU.1 is required for the development of mature myeloid and lymphoid cells. Due to this essential role and the importance of PU.1 in regulating several signature markers of lymphoid progenitors, its precise function in early lymphopoiesis has been difficult to define. Here, we demonstrate that PU.1 was required for efficient generation of lymphoid-primed multipotent progenitors (LMPPs) from hematopoietic stem cells and was essential for the subsequent formation of common lymphoid progenitors (CLPs). By contrast, further differentiation into the B-cell lineage was independent of PU.1. Examination of the transcriptional changes in conditional progenitors revealed that PU.1 activates lymphoid genes in LMPPs, while repressing genes normally expressed in neutrophils. These data identify PU.1 as a critical regulator of lymphoid priming and the transition between LMPPs and CLPs.

## Introduction

Hematopoietic stem cells (HSCs) are responsible for the development of all mature blood cell types. HSCs are found within the lineage (Lin)^−^Sca-1^+^c-Kit^+^ (LSK) population of the bone marrow (BM) and are identified within the LSK population as CD150^+^CD48^−^ cells [reviewed by Wilson et al. (1)]. The LSK population also includes lymphoid-primed multipotent progenitors (LMPPs) whose potential is skewed toward lymphocyte and myeloid differentiation (2). LMPPs are defined by a characteristically high cell surface concentration of Flt3 and express of a number of lymphoid transcripts, a process termed lymphoid priming. One of the genes subject to lineage priming is *Rag1*. Indeed the expression of a GFP reporter expressed from the *Rag1* locus can be used to identify a population termed the early lymphoid progenitor (ELP) that overlaps with the LMPP (3). The common lymphoid progenitor (CLP) is developmentally downstream of the LMPP and its potential appears largely restricted to the lymphoid lineages, *in vivo*, if not *in vitro* (4–6). CLPs upregulate expression of IL-7Rα, while maintaining Flt3 and Rag1/GFP (7, 8). Signaling through both, Flt3 and IL-7Rα, is required for development to the B cell progenitor stages (9). CLPs can be further divided through the expression of Ly6D into a true “all lymphocyte progenitor” (ALP, Ly6D^−^), which can give rise to all lymphocytic lineages, and a “B cell biased lymphocyte progenitor” (BLP, Ly6D^+^) (5, 7). BLPs differentiate directly into committed B cells through the concerted activity of E2A, EBF1, and Pax5 (10).

PU.1, encoded by the *Spi1* gene, has long been implicated as a key regulator of the cell fate decisions between the myeloid and lymphoid lineages (11–13). PU.1 concentration is highest in myeloid cells where it functions as a pioneer factor to broadly promote lineage-specific gene expression (14). PU.1 expression is reduced approximately 10-fold early during B-lymphopoiesis, and this low expression is maintained throughout the B cell differentiation process (15, 16). This change in PU.1 concentration is driven at least in part by a positive feedback loop that lengthens cell cycle duration, thus allowing accumulation of PU.1 protein in myeloid cells (17). The appropriate regulation of PU.1 expression is key to the lineage commitment process as deregulation of PU.1 leads in certain lineages to developmental blockade and can result in leukemia formation (18–22). The distinct concentrations of PU.1 in myeloid and lymphoid progenitors are thought to differentially activate a gene regulatory network involving PU.1, Ikaros, and secondary determinants such as Egr1 and Gfi1 (23–25). In this model, low PU.1 is achieved through the activity of Ikaros and Gfi1, resulting in the activation of EBF and the B cell program. This regulatory network is by no means complete, as other factors including E2A (26), Myb (27), and Mef2c (28) have also been implicated in the priming and differentiation of lymphoid progenitors in the BM.

PU.1-deficient embryos or adult mice conditionally deficient for PU.1 in HSCs lack mature lymphocytes (29). However, the determination as to when in lymphoid development PU.1 is required has been complicated by the regulation of several of the key diagnostic markers for LMPPs and CLPs (Flt3 and IL-7Rα) by PU.1 (12, 30, 31). Interestingly, conditional inactivation of PU.1 downstream of CLPs (by an *in vitro* retroviral transduction approach) (32) or B cells by CD19-Cre allows B cell development to proceed (33, 34), suggesting that the window of requirement for PU.1 is between the HSC and CLP stages. To address this issue directly, we have generated PU.1-deficient HSCs that also carry the Rag1/GFP reporter allele, thus enabling us to unambiguously identify LMPPs and CLPs without PU.1, while Rag1/Cre enabled the deletion of PU.1 in CLPs.

## Materials and Methods

### Mice

The *Spi1*^gfp^ (floxed exon 5 of *Spi1* and a GFP knockin into the 3′ untranslated region) (16), *Spi1*^fl/−^*MxCre*^+^ (35), *Rag1*^gfp/+^ (36), *Rag1*^cre^*^/+^* (37), and *Rosa26*-CreER^T2^ (38) mice have been previously described. For conditional inactivation of PU.1, *Spi1*^fl/−^ or *Spi1*^fl/fl^*MxCre*^+^ mice were injected intraperitoneal (i.p.) with 5 µg/g body weight of polyIC (GE Healthcare) twice at 3-day intervals. Mice were analyzed 14, 28, and 42 days after the first polyIC injection. Experimental mice were used at 6–12 weeks of age and maintained on a C57Bl/6 background.

### Preparation of Hematopoietic Progenitors

Hematopoietic cells were flushed from the tibia and femur of both legs. To enrich for hematopoietic progenitor populations in the BM, antibodies to the following surface molecules were used immunomagnetic bead depletion of lineage (Lin) marker-positive BM cells: CD2 (RM2-1), CD3 (KT-3.1), CD8 (53-6.7), CD11b/MAC-1 (M1-70), Gr-1 (RA6-8C5), B220 (RA3-6B2), and erythrocyte (TER119). Lin^+^ cells were exposed to BioMag goat anti-rat IgG beads (Qiagen) and depleted with a Dynal MPC-L magnet (Invitrogen). Lin^−^ BM cells were stained with labeled antibodies as described below.

### Flow Cytometry

The following anti-mouse mAbs were used: Sca-1 (E13161.7, produced in house), c-Kit (ACK2, produced in house), Flt3 (A2F10.1; BD Pharmingen), IL-7Rα (B12-1; eBioscience, Bioof), Ly6C (5075-3.6), Ly6D (49-H4, BD Pharmingen), CD19 (ID3; BD Pharmingen, eBioscience), B220 (RA3-6B2; BD Pharmingen, eBioscience), IgM (331.12, BD Pharmingen), NK1.1 (PK136, BD Pharmingen), CD49b (HMα2, BD Pharmingen), TCRβ (H57-597, BD Pharmingen), CD45.1 (A20, eBioscience), and CD34 (RAM34; BD Pharmingen). Anti-rat immunoglobulin-phycoerythrin and PECy7-streptavidin (BD Pharmingen) were used as secondary detection reagents.

Single cell suspensions were prepared in balanced salt solution with 2% (v/v) fetal calf serum. Cell staining was on ice for 30 min with fluorescent or biotin conjugated antibodies and the samples were processed on an LSRII or LSRFortessa flow cytometer (BD Biosciences). Propidium iodide exclusion was used to determine cell viability. Data were analyzed using FlowJo software (Treestar Inc.).

### RNA Isolation, Amplification From LSK Cells, and Array Analysis

Total RNA was isolated from LSK cells of PU.1 conditional deleted and wild-type animals (three pools of 15–20 mice treated with 5 µg/g of body weight polyIC 14 days previously) using RNeasy kits (Qiagen). RNA was amplified with the Illumina Total Prep RNA Amplification Kit (Ambion) and quantity and quality assessed using the Agilent Bioanalyzer 2100. Labeled cRNA was hybridized to Illumina MouseWG-6 V 2.0 Expression BeadChips at the Australian Genome Research Facility, Melbourne.

The resulting arrays were analyzed in R using the Bioconductor package *limma* (39). Raw intensities were normalized by using the neqc function, which performs background and quantile normalization using control probes (40). Probes not detected in any sample were removed (detection *p* value < 0.05). Pairwise comparisons used linear modeling and empirical Bayes moderated *t* statistics (41). The false discovery rate (FDR) was controlled by the Benjamini–Hochberg algorithm. Differentially expressed probes had an FDR of <0.05. Multi-dimensional scaling (MDS) plot was produced using expression data for wild-type progenitor and stem cell populations obtained from http://haemosphere.org (42) using plotMDS function in *limma* using the top 500 differentially expressed genes. Lineage-specific gene sets obtained from http://haemosphere.org (42) were used in gene set tests. *p* Values were obtained with rotation gene set testing (ROAST) using the mroast in *limma*, Benjamini–Hochberg correction for multiple testing and 9,999 rotations. Barcode plots were made using the barcodeplot function of the *limma* package.

Gene ontology analyses were obtained from PANTHER Classification System version 11.0 (43). Statistical overrepresentation test was performed on activated and repressed genes, using PANTHER GO-Slim Biological Process. Only results with *p* value <0.05 and positive fold enrichment >1 are displayed.

### Quantitative Real-Time RT-PCR

Total RNA was isolated from purified cells using TRIzol (Invitrogen). cDNA synthesis used iScript Reverse Transcription Supermix (Bio-Rad). Quantification of gene expression was performed in triplicate with QuantiTect SYBR Green PCR kit (Qiagen) on a C1000 Thermal Cycler (Bio-Rad). The primers are listed in Table S1 in Supplementary Material.

### *In Vitro* Clonogenic Assays

To determine the progenitor frequencies, sorted populations were seeded in limiting dilution on OP9 stromal cells in media containing 2% IL-7 supernatant and 5 ng/ml Flt3L. 350 nM 4-hydroxytamoxifen (4OT) was added at day 0. The media was diluted 1:3 on day 1 to reduce any cytotoxic effect of the 4OT. Cells were scored after 7–10 days as described (44). The clonogenic frequency was calculated using the ELDA software (45).

### Statistical Analysis

Statistical analysis used the GraphPad Prism software. For the non-microarray data, sample size analysis indicates that the power to detect a twofold change with a type error of <5% and power of 90% confidence and an SD of 20% of the mean is 4 mice/genotype. We used between 5 and 14 mice/genotype over two to three independent experiments as outlined in the figure legends. Paired or unpaired, two-tailed, Student’s *t*-test for two samples with equal variance was used as appropriate.

## Results

### PU.1 Expression Peaks in LMPPs During Early Lymphopoiesis

Previous studies have reported that LSK cells and CLPs express relatively high amounts of PU.1 (15, 16, 46). However, fine mapping of PU.1 expression in the LMPPs and the ALP and BLP fractions of the CLP has not been reported. In order to quantify the expression of *Spi1* mRNA in these purified progenitor populations, we utilized mice homozygous for a PU.1/GFP reporter allele that does not impact on PU.1 function (16) and correlates extremely well with PU.1 protein (17). Flow cytometric analysis revealed robust PU.1/GFP expression in all multipotent progenitors (MPPs) with lymphoid potential, with expression peaking in LMPPs (Figures S1A–C in Supplementary Material). PU.1 expression reduced slightly in ALPs and BLPs, before dropping to the characteristic low expression in pro-B-cells and absence in NK cells. The expression of PU.1/GFP matched closely the expression of *Spi1* mRNA in the corresponding populations in the ImmGen^1^ (Figure S1D in Supplementary Material) and Gene Expression Commons^2^ [Figure S1E in Supplementary Material (47)] databases. Thus within the lymphoid developmental pathway, PU.1 expression peaks at the LMPP stage.

### Analysis of PU.1-Deficient Lymphopoiesis Using Rag1/GFP

Conventional fractionation of the lymphoid progenitor compartment relies heavily on cell surface markers Flt3 and IL-7R. We have previously shown that the gene encoding Flt3, the defining marker of LMPPs within the LSK pool and expressed on all CLPs, is an obligate PU.1 target gene [(30) and confirmed in all BM progenitors in Figures S2A–D in Supplementary Material], while other have shown that PU.1 regulates the gene encoding the IL-7Rα chain (12), the defining marker of CLPs. To assess the impact of PU.1 inactivation for lymphoid progenitors independently of Flt3 and IL-7R, we have utilized an alternative strategy to identify these populations using Rag1/GFP (36). Rag1/GFP expressing cells within the LSK population define ELPs (3–6% within the LSK cells), which has been demonstrated to be the true lymphoid-primed subset of the LMPP (3). Despite being lymphoid-primed, virtually all ELPs co-express CD34 and Flt3 (Figure S3A in Supplementary Material) and only a low proportion (7.3 ± 1.2%) expressed cell surface IL-7R. Similarly, there is a high degree of surface marker expression overlap between conventionally defined CLPs (Lin^−^Sca-1^+^c-Kit^int^Flt3^+^IL-7Rα^+^) and Lin^−^Sca-1^+^c-Kit^int^Rag1/GFP^+^ CLPs (Figure S3B in Supplementary Material) (7, 8). Importantly, our previous RNAseq analysis has shown that *Rag1* expression is independent of PU.1 in B cell progenitors (48). Thus Rag1/GFP can be used as a reliable surrogate for Flt3 and IL-7R in the identification of LMPPs and CLPs in the absence of PU.1.

**Figure 1:**
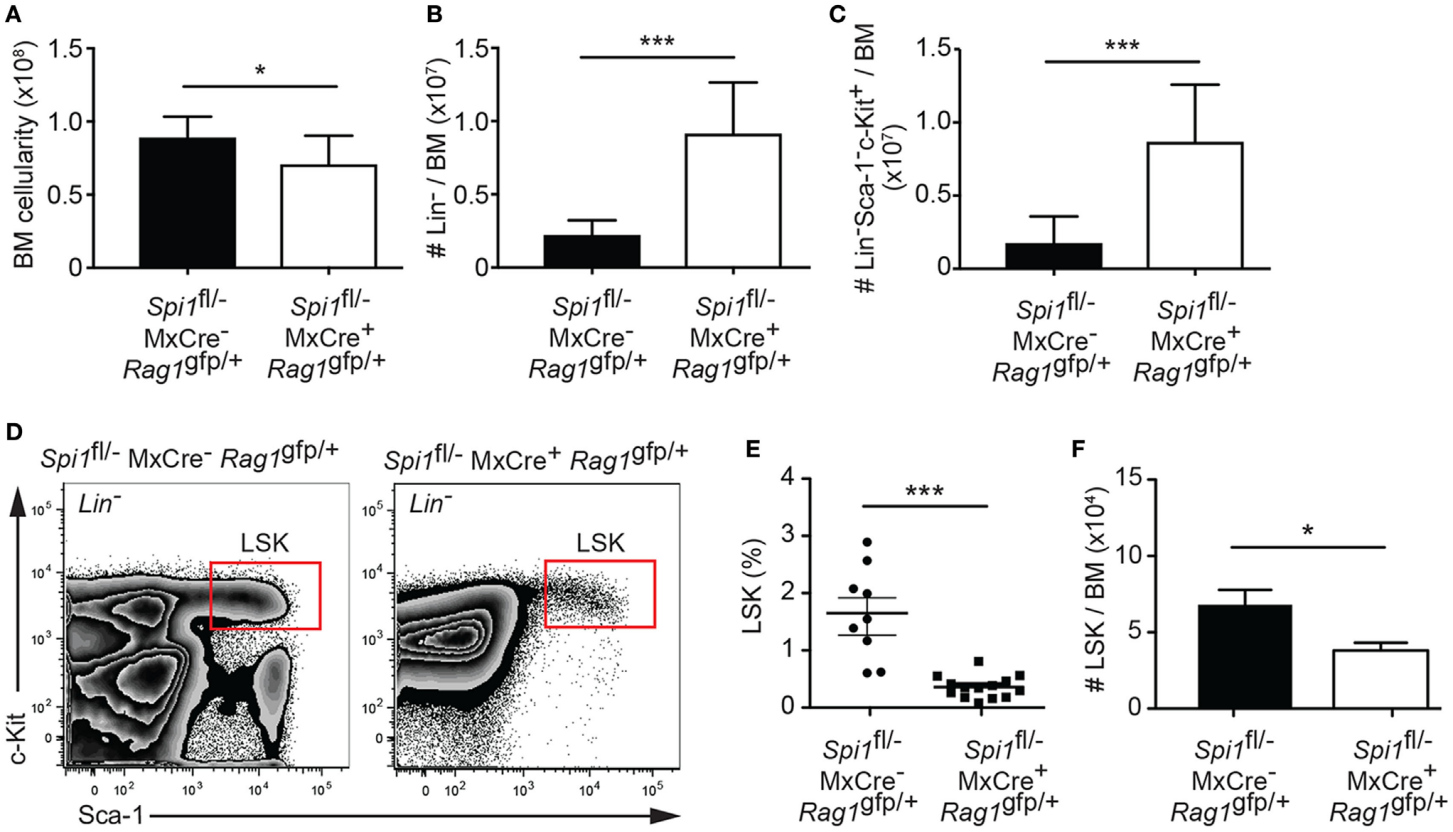
Reduced numbers of hematopoietic progenitors from PU.1 conditionally deficient bone marrow (BM). *Spi1*^fl/−^MxCre^−^*Rag1*^gfp/+^ and *Spi1*^fl/−^MxCre^+^*Rag1*^gfp/+^ mice were injected with polyIC on days 0 and 3, and analyzed by flow cytometry on day 14. Graph shows **(A)** the absolute cell numbers in the BM (2× femur and tibia), **(B)** the number of Lin^−^ cells, and **(C)** the number of Lin^−^Sca-1^−^c-Kit^+^ cells in BM preparation. **(D)** Representative flow cytometry plot of Lin^−^ BM preparations from the mice of indicated genotypes. Boxes indicate the position of the LSK populations. **(E)** Graph shows the proportion of LSK cells in Lin^−^ BM preparation. Each dot represents an individual BM sample. Horizontal line shows the mean ± SD. **(F)** Absolute LSK cell numbers in the BM. Data in the graphs are the mean cell number ± SD from between 9 and 14 mice per genotype. *p* Values compare the indicated groups using an unpaired *t*-test. *p < 0.05, ***p < 0.001.

To investigate the requirement of PU.1 in hematopoietic progenitors, we have administered polyIC into *Spi1*^fl/−^*MxCre*^−^*Rag1*^gfp/+^ or *Spi1*^fl/−^*MxCre^+^Rag1*^gfp/+^ mice, to generate heterozygous and null cells, respectively. PolyIC activates the MxCre transgene *via* the type I interferon pathway (49), a process that transiently perturbs BM hematopoiesis, although we have previously shown that control hematopoiesis returns to the steady state within 14 days of the treatment (35). The mouse *Spi1* and *Rag1* genes are closely linked on chromosome 2. Due to inefficient meiotic crossover, we were unable to any offspring of *Spi1*^fl/fl^ x *Rag1*^gfp/+^ crosses carrying the *Spi1*^fl^ and *Rag1*^gfp^ alleles on the same chromosome. We did, however, obtain a single recombination event from a *Spi1*^fl/−^ and *Rag1*^gfp/+^ cross that resulted in linkage between the *Spi1*^−^ (null) allele and *Rag1*^gfp^. Thus for this technical reason, we compared *Spi1*^fl/−^*MxCre*^−^*Rag1*^gfp/+^ (control) and *Spi1*^fl/−^*MxCre^+^Rag1*^gfp/+^ (experimental) genotypes in each experiment. At this time point, PU.1 inactivation resulted in a modest reduction in overall BM cellularity, but a markedly increased proportion of these cells were Lin^−^ progenitors (Figures 1A,B). The increased Lin^−^ compartment in *Spi1*^fl/−^*MxCre^+^Rag1*^gfp/+^ mice was due to an increase in the proportion of Sca-1^−^c-Kit^+^ myeloid progenitors as we have previously shown [Figures 1C,D (35)]. This analysis also revealed a highly significant reduction in the proportion of LSK cells without PU.1 (Figures 1D,E). However, when absolute cellularity was determined, it became apparent that loss of PU.1 resulted in a twofold reduction in LSK cells (Figure 1F). Thus the loss of PU.1 had a relatively mild impact on the frequency of LSK cells.

**Figure 2:**
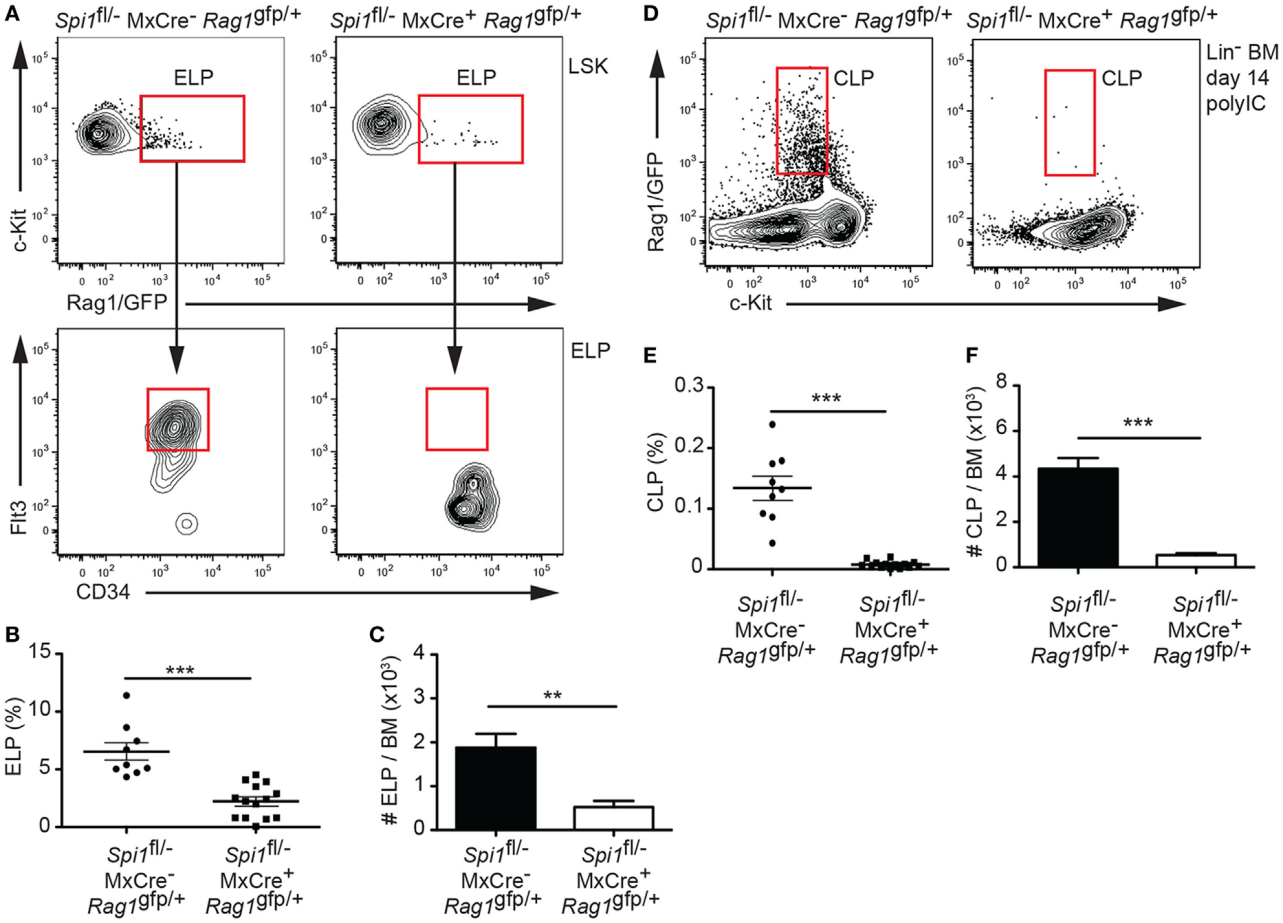
Absence of lymphoid progenitors from PU.1 conditionally deficient bone marrow (BM). *Spi1*^fl/−^MxCre^−^*Rag1*^gfp/+^ and *Spi1*^fl/−^MxCre^+^*Rag1*^gfp/+^ mice were injected with polyIC on days 0 and 3, and analyzed by flow cytometry on day 14. **(A)** Upper plots, representative flow cytometry plot of the LSK populations, gated as in Figure 1D, from the mice of indicated genotypes. Boxes indicate the position of the Rag1/GFP^+^ early lymphoid progenitor (ELP) populations. Lower plots, ELPs express Flt3 and CD34 in control *Spi1*^fl/−^MxCre^−^*Rag1*^gfp/+^ ELPs, but not in the *Spi1*^fl/−^MxCre^+^*Rag1*^gfp/+^ cells, confirming the inactivation of PU.1. **(B)** Graph shows the proportion of ELPs cells in the LSK cell gate. Each dot represents an individual BM sample. Horizontal line shows the mean ± SD. **(C)** Absolute ELP numbers in the BM. **(D)** Representative flow cytometry plot of Lin^−^ BM preparations from the mice of indicated genotypes. Boxes indicate the position of the common lymphoid progenitor (CLP)-equivalent populations using Rag1/GFP. **(E)** Graph shows the proportion of Rag1/GFP^+^ CLPs cells in the Lin^−^ gate. Each dot represents an individual BM sample. Horizontal line shows the mean ± SD. **(F)** Absolute Rag1/GFP^+^ CLP numbers in the BM. Data in panels **(C,F)** are the mean cell number ± SD from between 9 and 14 mice per genotype. *p* Values compare the indicated groups using an unpaired *t*-test. **p < 0.01, ***p < 0.001.

**Figure 3:**
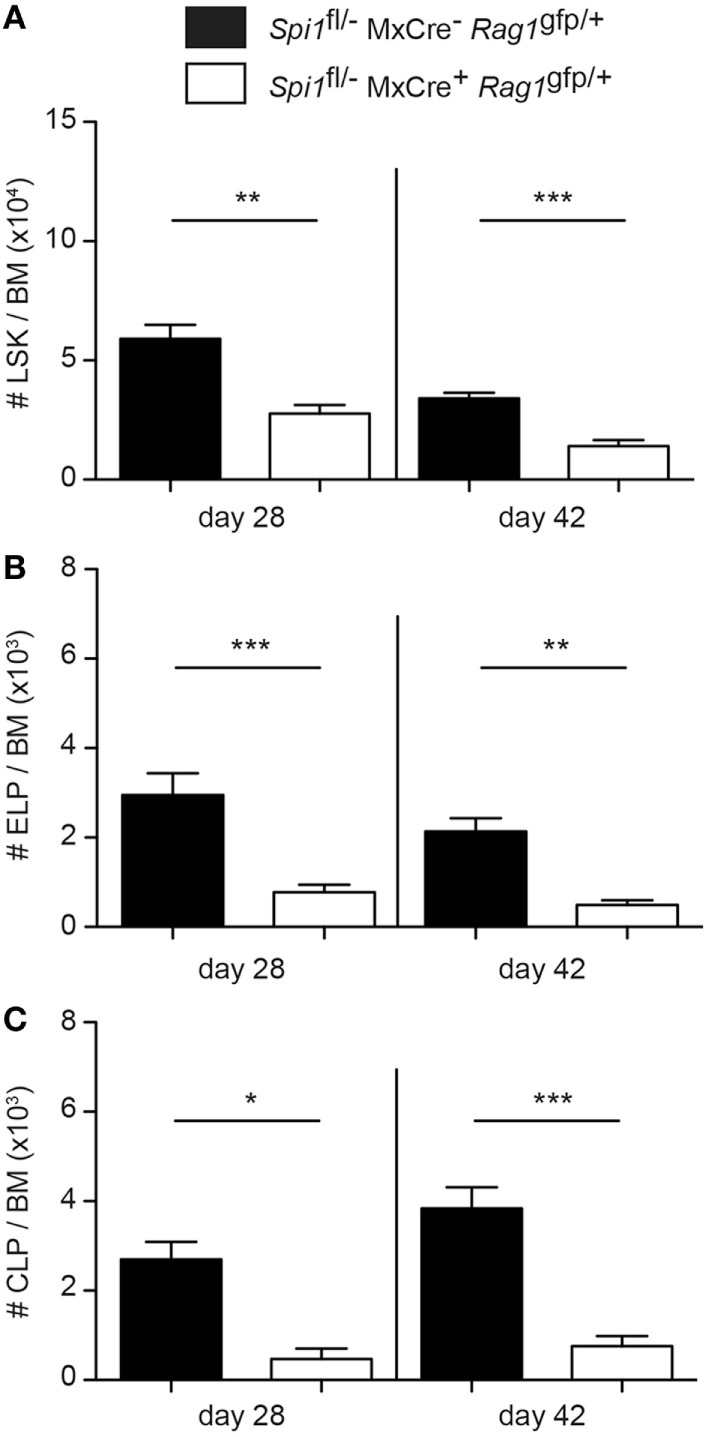
Sustained reduction in lymphoid progenitors in PU.1 conditionally deficient bone marrow (BM). *Spi1*^fl/−^MxCre^−^*Rag1*^gfp/+^ and *Spi1*^fl/−^MxCre^+^*Rag1*^gfp/+^ mice were injected with polyIC on days 0 and 3, and analyzed by flow cytometry on days 28 and 42. Total numbers of **(A)** LSK cells, **(B)** Rag1/GFP^+^ early lymphoid progenitors, and **(C)** Rag1/GFP^+^ common lymphoid progenitors (CLPs) in the BM of indicated genotypes were calculated. The data are mean ± SD from between 5 and 8 mice per genotype. *p* Values compare the indicated groups using an unpaired *t*-test. *p < 0.05, **p < 0.01, ***p < 0.001.

Analysis of the ELP fraction of the LSK revealed that PU.1 inactivation resulted in a significant decrease in the proportion and frequency of ELPs (Figures 2A–C). Importantly, the remaining ELPs lacked Flt3, suggesting that they were PU.1 deficient (Figure 2A). By contrast, analysis of the Rag1/GFP^+^ CLP fraction showed an absence of CLPs in the BM, indicating that PU.1 was essential for the formation of these cells (Figures 2D–F). Although the experiments described in Figures 1 and 2 were conducted 14 days after the initial polyIC treatment, we believe that this represented the steady-state situation as analysis of the frequency of the LSK, ELP, and CLP populations at days 28 and 42 after polyIC exposure produced very similar results (Figures 3A–C). Taken together these data indicate that the requirement for PU.1 progressively increases as lymphoid progenitors transition between the LSK and CLP compartments.

### PU.1 Is Dispensable From the CLP Stage of Development

**Figure 4:**
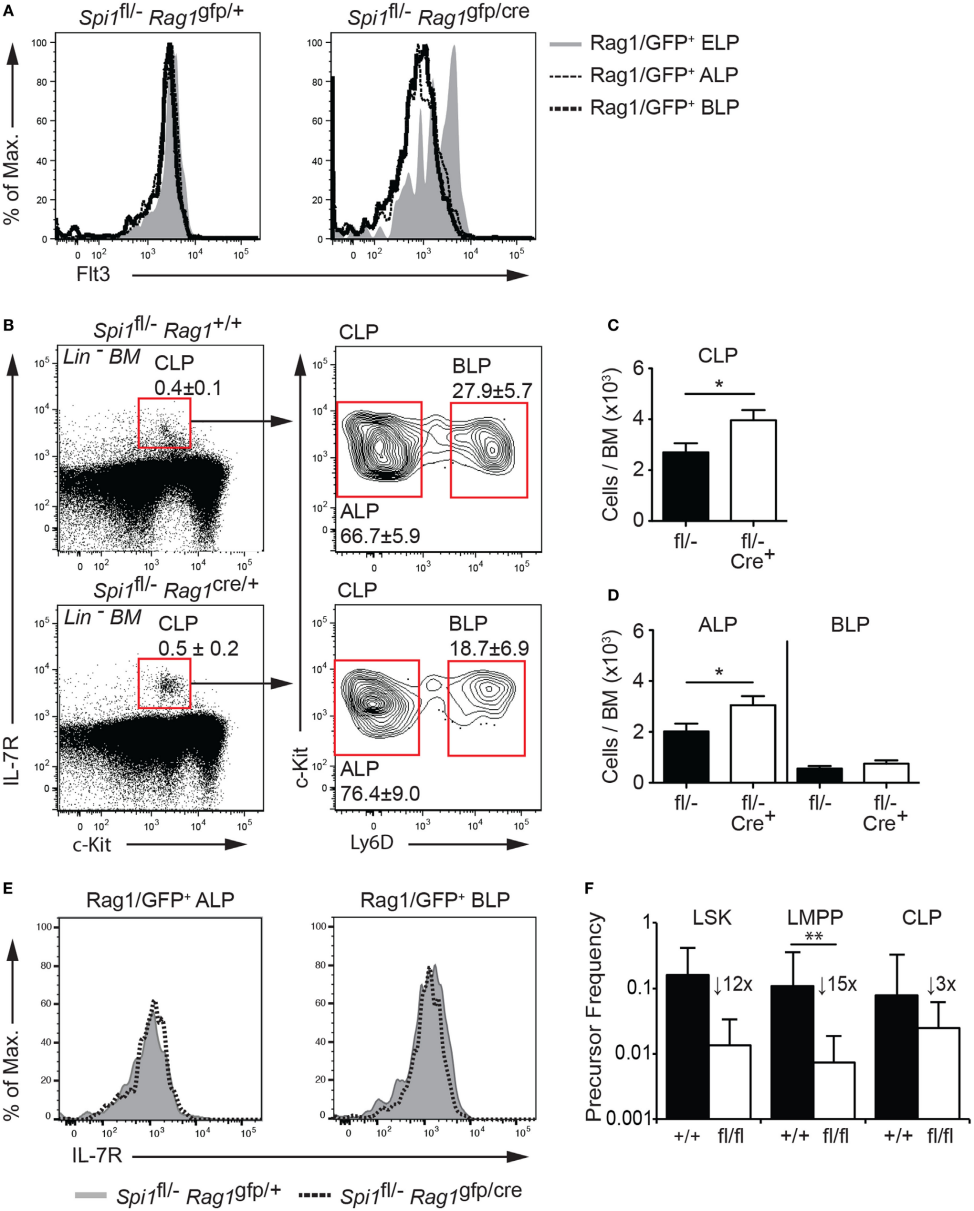
PU.1 is not required for the persistence of common lymphoid progenitors (CLPs). **(A)** Lineage-depleted (Lin^−^) bone marrow (BM) was isolated from *Spi1*^fl/−^*Rag1*^+/gfp^ and *Spi1*^fl/−^*Rag1*^cre/gfp^ mice and Rag1/GFP^+^ early lymphoid progenitors (ELPs), all lymphocyte progenitors (ALPs), and biased lymphocyte progenitors (BLPs) of each indicated genotype were assessed for Flt3. **(B–D)** Lin^−^ BM from *Spi1*^fl/−^*Rag1*^+/+^ and *Spi1*^fl/−^*Rag1*^cre/+^ mice were analyzed for the **(B)** frequency and **(C)** number of CLPs and **(D)** number of ALPs and BLPs. Boxes show the position of gating for the cell type being analyzed. **(E)** Lineage-depleted (Lin^−^) BM was isolated from *Spi1*^fl/−^*Rag1*^+/gfp^ and *Spi1*^fl/−^*Rag1*^cre/gfp^ mice and Rag1/GFP^+^ ALPs and BLPs of each indicated genotype were assessed for IL-7R. Data in panels **(A,E)** are representative of two experiments each consisting of two mice per genotype. Data in panels **(B–D)** are the mean ± SD from between 9 and 13 mice per genotype. **(F)** Sorted LSK cells, lymphoid-primed multipotent progenitors (LMPPs), and CLPs from *Spi1*^fl/fl^CreER^T2^ and control *Spi1*^+/+^CreER^T2^ mice and cultured in limiting dilution with OP9 stromal cells in the presence of IL-7 and Flt3L for 7–10 days. 350 nM 4-hydroxytamoxifen was added to all cultures on day 1 and diluted threefold after 24 h. The mean clonogenic frequency ±5% confidence interval of two experiments each with triplicate measurements is shown. *p* Values compare the indicated groups using an unpaired *t*-test. *p < 0.05, **p < 0.01.

The data described above demonstrated that PU.1 was required for the development of Rag1/GFP^+^ CLPs from LMPPs, although whether PU.1 has an essential function in CLPs was unclear. A previous study showed that removal of PU.1 in CLPs was compatible with B cell development (32), however, as that study required the CLPs to be cultured for 2 days in B cell promoting conditions prior to Cre activation and PU.1 removal, it remained to be determined if PU.1 was required at the CLP stage of differentiation *in vivo*. To address this question, we crossed the *Spi1*^fl/−^ allele to *Rag1*^cre^*^/+^* knockin mice (note that *Rag1*^cre/gfp^ mice lacked lymphocytes beyond the pro-B/T cell stage due to the absence of Rag1 function but allowed the tracking of the Rag1/GFP^+^ CLPs). In this case, we achieved the desired meiotic crossover, producing a copy of chromosome 2 carrying *Spi1*^fl^ and *Rag1*^cre^. Rag1/Cre has been shown to initiate deletion of a floxed allele in the LSK compartment (37), has substantial but not complete activity at the CLP stage (50) and complete deletion in pro-B and pro-T cells (51). To check for the efficacy of Rag1/Cre in lymphoid progenitors, we assessed Flt3 expression in Lin^−^ BM from *Spi1*^fl/−^*Rag1*^cre/gfp^ and control *Spi1*^fl/−^*Rag1*^+/gfp^ mice as a surrogate marker for PU.1 deletion. We observed a marked reduction of Flt3 expression in ALPs and BLPs within the CLP compartment (Figure 4A), suggesting that *Spi1* deletion occurred efficiently in those cells, while as expected the majority of ELPs maintained Flt3 expression (Figure 4A). The fluorescence intensity of the IL-7R was similar between the genotypes suggesting that PU.1 was not required for *Il7r* expression at the ALP or BLP stages of development (Figure 4E).

In keeping with the onset of *Spi1* inactivation at the CLP stage, the proportion and absolute number of LSK cells and LMPPs in the BM of *Spi1*^fl/−^*Rag1*^cre^*^/+^* and control *Spi1*^fl/−^*Rag1^+/+^* mice was equivalent (Figures S4A–D in Supplementary Material). Closer examination of the CLP compartment revealed a statistically significant increase in total CLPs in the absence of PU.1, which resulted from a similarly increased proportion and frequency of ALPs (Figures 4B–D). The frequency of BLPs, the earliest B cell progenitor and lineage committed pro and pre-B cells were equivalent between the genotypes suggesting that PU.1 activity appears largely dispensable for the differentiation of CLPs into mature lymphoid lineages *in vivo* (Figures 4B–D and data not shown).

The conclusion that PU.1 was not required beyond the transition to the CLP stage was supported by *in vitro* clonogenic assays. These experiments utilized *Spi1*^fl/fl^ mice crossed to the *Rosa26-*CreER^T2^ (*Spi1*^fl/fl^CreER^T2^) allele that allows 4-hydroxytamoxifen (Tam) mediated induction of Cre activity *in vitro*. LSK cells, LMPPs and CLPs were isolated from the BM of *Spi1*^fl/fl^CreER^T2^ and control *Spi1*^+/+^CreER^T2^ mice and cultured in limiting dilution with OP9 stromal cells, plus Flt3L, and IL-7 for 7–10 days. All cultures were exposed to 350 nM Tam for the first day. As expected, all progenitor fractions from the control PU.1^+/+^CreER^T2^ gave rise to B cells colonies at a cloning frequency of ~10% (Figure 4F). LSK cells and LMPPs from *Spi1*^fl/fl^CreER^T2^ mice showed marked reduction in B cell clonogenic potential (11.9-fold reduction for HSCs and 14.7-fold reduction for LMPPs) compared to controls. By contrast, CLPs from the same mice were much less sensitive to PU.1 loss (threefold reduction compared to controls) a finding in agreement with a previous study (32). Collectively, these results demonstrate that PU.1 is required for the LMPP to CLP transition and suggested no obligate role for this factor at subsequent points in early B cell development.

### PU.1 Activates Lymphoid Associated Genes in MPP Cells

**Figure 5:**
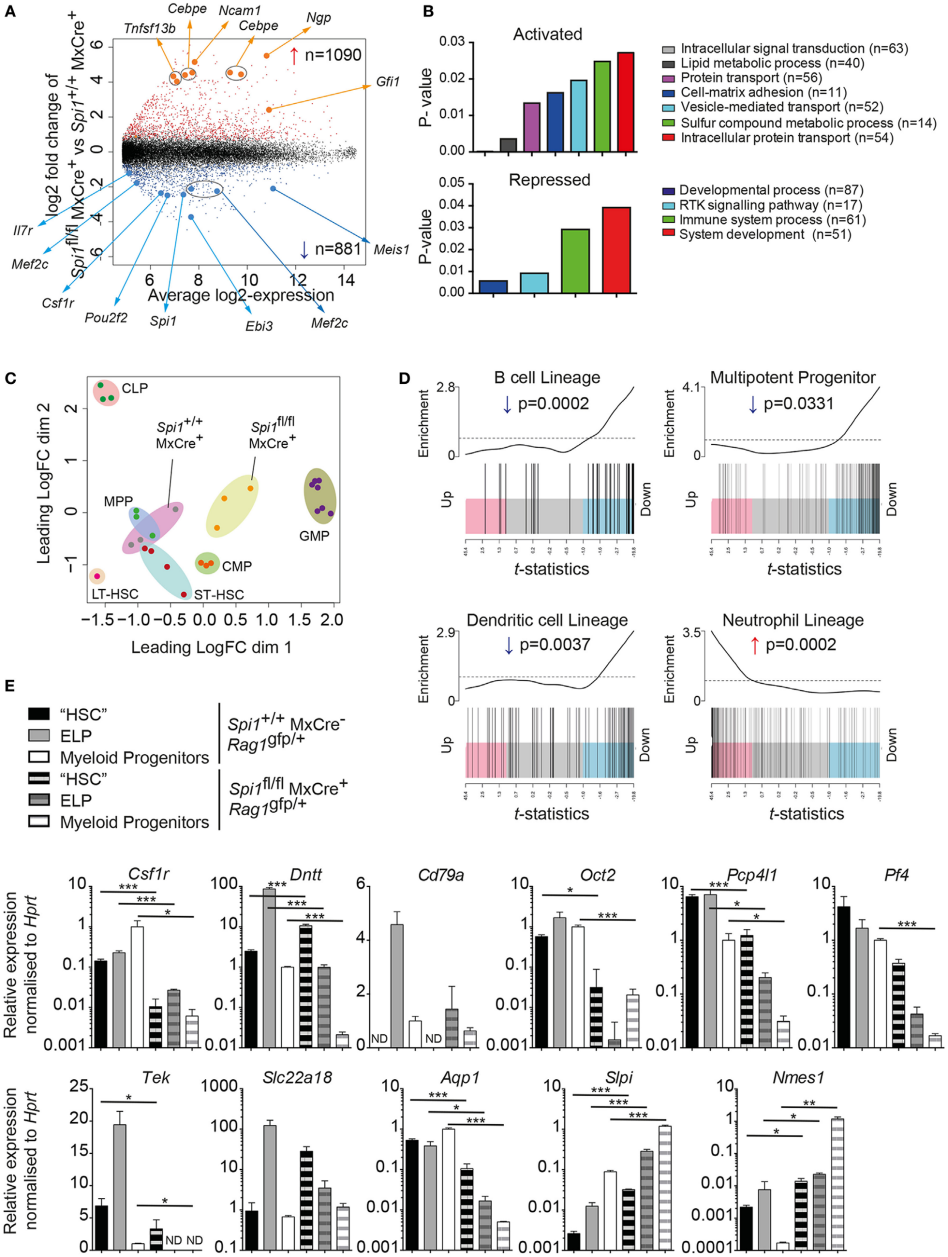
PU.1 is required for lymphoid gene priming in LSK cells. **(A–D)** *Spi1*^+/+^MxCre^+^ and *Spi1*^fl/fl^MxCre^+^ mice were injected with polyIC on days 0 and 3, and LSK cells were isolated by flow cytometry on day 14. Three independent samples of LSK cells from each genotype (each a pool of 15–20 individual mice) were analyzed by gene expression profiling. **(A)** Scatter plot of differential expression. Genes with significantly increased (red) or decreased (blue) in the absence of PU.1 are indicated (false discovery rate < 0.05). The number of differentially expressed genes is indicated. Position of probes corresponding to genes of interest is highlighted. **(B)** Bar charts showing gene ontology classification of activated and repressed genes by Panther GO-Slim biological process dataset (*p* value < 0.05 and fold enrichment > +1). The number of differentially expressed genes in each GO category are indicated **(C)** MDS plot of top 500 differentially regulated genes to demonstrate the relatedness of gene profiles of the indicated populations. Abbreviations: LT-HSC, long term-hematopoietic stem cell; ST-HSC, short term-HSC; MPP, multipotent progenitor; CLP, common lymphoid progenitor; CMP, common myeloid progenitor; GMP, granulocyte macrophage progenitor; MDS, multi-dimensional scaling. Each dot represents the indicated dataset. Close clustering of biological replicates of each cell type is highlighted by shaded ovals. **(D)** Barcode plot of B cell, MPP, dendritic cell, and neutrophil gene signatures compared to gene expression changes after *Spi1* deletion in LSK cells. Genes (shaded rectangles; horizontally ranked by moderated *t*-statistic) upregulated (pink; *t* > 1), downregulated (blue; *t* < −1) or not altered (gray) in *Spi1*^+/+^MxCre^+^ compared to *Spi1*^fl/fl^MxCre^+^ LSK cells. Vertical black lines indicate the genes from the indicated signatures. Top, worm shows relative local enrichment of signature genes in each part of the plot with the dotted horizontal line indicating neutral enrichment. Data of the indicated populations in panels **(C,D)** were obtained from http://haemosphere.org (42). **(E)** Quantitative real-time RT-PCR of indicated lymphoid and myeloid associated genes to confirm differential gene expression. *Spi1*^fl/fl^MxCre^−^*Rag1*^gfp/+^ and *Spi1*^fl/fl^MxCre^+^*Rag1*^gfp/+^ mice were injected with polyIC on days 0 and 3, and Rag1/GFP^−^ LSK cells (“HSC”), Rag1/GFP^+^ LSK cells [early lymphoid progenitor (ELP)], and Lin^−^c-kit^+^Sca-1^−^ (myeloid progenitor cells) were isolated by flow cytometry on day 14 (as described in Figure S5 in Supplementary Material). Expression values are the mean ± SD of three independent experiments and are normalized to *Hprt*. *p* Values compare the indicated groups using an unpaired *t*-test. *p < 0.05, **p < 0.01, ***p < 0.001.

In order to examine the transcriptional roles of PU.1 in early progenitor populations, we purified wild type (*Spi1*^+/+^*MxCre*^+^) and PU.1-deficient (*Spi1*^flfl^*MxCre*^+^) LSK cells from mice treated 14 days previously with polyIC, and subjected the cells for gene expression profiling by oligonucleotide microarray. Analysis of this data revealed 1,971 differentially expressed transcripts, including 1,090 whose expression increased in the absence of PU.1, such as the transcription factors *Gfi1* and *Cebpe*, and 881 genes that required PU.1 for full expression including the known targets *Csf1r, Il7r*, and *Mef2c* (FDR < 0.05, Figure 5A). *Ebf1*, another target of PU.1 in pro-B cells (52), was not differentially expressed between the wild type and PU.1-deficient hematopoietic progenitors. We confirmed the efficient inactivation of *Spi1* as the signal detected from the oligonucleotide probe corresponding to the floxed *Spi1* exon 5 decreased >80% in each sample (Figure 5A). Pathway analysis revealed that the activated genes encoded proteins mostly involved in signaling, adhesion, and metabolic processes, while the repressed genes were associated with developmental and immune processes (Figure 5B).

To explore the relationships between the wild type and PU.1-deficient hematopoietic progenitors in an unbiased way, we measured the transcriptional distance between any pair of expression profiles. This analysis used the *leading fold change*, defined as the average fold change for the 500 genes most different between the samples. Data are shown on an MDS plot where the distances on the plot correspond to log2-leading fold change (Figure 5C). This analysis revealed that PU.1-deficient LSK cells clustered closely to the myeloid progenitors (common myeloid progenitor and granulocyte macrophage progenitor), in contrast to the control LSK cells that clustered adjacent to the defined HSC (LT- and ST-) and MPP populations (Figure 5C). This conclusion was further supported by cell lineage-specific gene set testing showing that repressed genes were enriched for the neutrophil lineage associated transcripts (Figures 5A,D), most likely representing the aberrant c-Kit^int^ population observed in the PU.1-deficient LSK cells [Figures 1C,D and (35)]. Most importantly, signatures of MPPs, B cell, and DC lineages were lost in the absence of PU.1 (Figure 5D).

To confirm the role for PU.1 in promoting the transcriptional priming of lymphoid genes in the MPPs, we analyzed the expression of some of the cohort of neutrophil and B cell specific genes identified in Figure 5D, in purified Rag1/GFP^+^ (ELP) and Rag1/GFP^−^ (“HSC”) LSK cells, as well as Lin^−^Sca-1^−^c-kit^+^ myeloid progenitors from the BM of *Spi1*^flfl^*MxCre*^+^*Rag1*^gfp/+^ (from a newly generated mouse line housing a meiotic recombination producing linked *Spi1*^fl^ and *Rag1*^gfp^) and control *Spi1*^+/+^*MxCre*^−^*Rag1*^gfp/+^ mice (Figure 5E; Figure S5 in Supplementary Material). The efficient deletion of *Spi1* was confirmed by the absence of Flt3 on the cell surface (Figure S5 in Supplementary Material), and again by the reduced mRNA for *Csf1r* (encoded for MCSF-R) whose expression is regulated by PU.1 (53) (Figure 5E). In agreement with the gene expression studies, we confirmed eight downregulated genes for the B cell lineage (*Dntt, Cd79a, Oct2, Pcp4l1, Pf4, Tek, Slc22a18*, and *Aqp1*) and two upregulated genes (*Slpi, Nmes1*) for neutrophil lineage in PU.1-deficient ELPs (Figure 5E). Together, these data demonstrate that PU.1 is broadly required to prime the expression of lymphoid genes and suppress some neutrophil genes in ELPs for subsequent formation into CLPs.

## Discussion

PU.1 is one of a small group of transcriptional regulators, including Ikaros, E2A, Mef2c, EBF1, Myb, and Pax5 that control the specification and commitment of lymphoid progenitors to the B cell pathway (54–56). Although, it has long been known that PU.1 is required for lymphocyte formation from either the fetal or adult HSCs, the function of PU.1 in lymphopoiesis and the exact point at which it is required has proven more elusive. Here, we demonstrate using conditional mutagenesis that PU.1 is required for efficient LMPP formation and for the subsequent differentiation to the CLP stage.

It has been proposed that the concentration of PU.1 in MPPs determines myeloid (high PU.1) or lymphoid (low PU.1) outcomes (13). Our analysis of *Spi1* transcription using a GFP reporter allele that did not impact on PU.1 function (16) suggests that *Spi1* was relatively uniformly expressed throughout early hematopoietic development, with expression peaking at the LMPP stage. These data mirrored closely the expression of *Spi1* mRNA in wild-type hematopoietic cells. Within the lymphoid developmental pathway PU.1 expression slowly declined but was not markedly downregulated until the pro-B cell (Fraction B) stage. By contrast, myeloid progenitors cells express an even higher concentration of PU.1 than that observed in LMPPs, which peaks in mature myeloid cells (13, 15–17, 19, 30, 57).

One of the limitations in mapping PU.1 function in early lymphopoiesis has been due to its role in the transcriptional regulation of the genes encoding Flt3 (30) and the α chain of the IL-7R (12), markers critical for defining the LMPP and CLP. Expression of Rag1/GFP in the LSK compartment defined ELPs that are the most lymphoid-primed component of the LMPP, while GFP expression in Lin^−^Sca-1^+^c-kit^int^ cells defined CLPs, independent of either Flt3 or IL-7R. Generation of PU.1-deficient hematopoietic progenitors expressing Rag1/GFP overcame this technical bottleneck, as the expression of *Rag1* is PU.1 independent in both pro-B cells and ALPs [(48) and data not shown]. This analysis revealed that PU.1 was required for the efficient production of LMPPs and was essential for CLP formation. The requirement was very stage specific as the removal of PU.1 using Rag1/Cre was compatible with CLP formation and B cell differentiation, in agreement with a previous study using a less direct approach (32). Thus, the critical role of PU.1 in lymphopoiesis occurs before the CLP stage of development by inducing lymphoid-specific genes and keeping myeloid genes in check at the LMPP stage. Although in the current study we have only addressed PU.1 function in adult BM lymphopoiesis, recent analysis of mice homozygous for a hypomorphic allele of *Spi1* [UREΔ/Δ (20)] demonstrated a concentration dependent function for PU.1 in controlling distinct waves of fetal and adult B-lymphopoiesis, suggesting additional complexities in the process (58). It should also be noted that the importance of PU.1 in some aspects of later B cell differentiation, such as at the pre- and mature B cell stages is masked by functional redundancy with the closely related gene, SpiB (59–61).

The phenotype arising from PU.1 deficiency, is broadly similar to that observed in mice lacking E2A (26), Mef2c (28), Ikaros (31, 62), and Myb (27), factors also required for the priming of lymphoid lineage genes in LSK cells. The links between PU.1 and the other members of this gene regulatory network are only partially understood. PU.1 directly regulates *Mef2c*, a factor that also regulates the myeloid versus lymphoid fate decision in hematopoietic progenitors and is essential for the formation of CLPs (28). It has been proposed that PU.1 concentration is determined by a regulatory circuit whereby activation of the lymphocyte-promoting factor Ikaros, itself an essential for lymphopoiesis, represses PU.1 expression either directly or *via* the induction of the repressor Gfi1 (25). In keeping with this concept, B cell development is impaired in the absence of Gfi1 and can be partially restored by the removal of one allele of *Spi1* or by shRNA-mediated knockdown of *Spi1*. Interestingly, *Gfi1* expression was increased in PU.1-deficient LSK cells, suggesting that PU.1 also functions at some level to repress *Gfi1* (Figure 5A). Similar counteracting networks are also known to result in multi-lineage priming and contribute to myeloid cell lineage determination (63). Together with the regulation of the two key cytokine receptors, Flt3 and IL-7R, it is likely that the regulation of these targets is sufficient to explain the important function of PU.1 in the transition from the LMPP to CLP stage of development.

## Availability of Data and Material

The microarray gene expression data are publicly available at the Gene Expression Omnibus (www.ncbi.nlm.nih.gov/geo), accession number GSE89642.

## Ethics Statement

This study was carried out in accordance with the recommendations of the Australian Code for the Care and Use of Animals for Scientific Purposes. The Walter and Eliza Hall Institute Animal Ethics Committee approved the protocols.

## Author Contributions

SP performed all experiments; CG performed the bioinformatics; SP, CG, SC, LW, and SN designed and analyzed the data; DH, NH, SC, LW, and SN supervised the research; SP and SN wrote the manuscript.

## Conflict of Interest Statement

The authors declare that the research was conducted in the absence of any commercial or financial relationships that could be construed as a potential conflict of interest.

## Footnotes

^1^www.immgen.org (Accessed: March 26, 2018).

^2^https://gexc.riken.jp/models/3/genes (Accessed: September 18, 2017).
