# Supplementary material for: PU.1 Is Required for the Developmental Progression of Multipotent Progenitors to Common Lymphoid Progenitors
Source: Front Immunol. 2018 Jun 11;9:1264. doi: 10.3389/fimmu.2018.01264 (PMC6005176; doi:10.3389/fimmu.2018.01264)
Supplement: Supplementary file 1 [file data_sheet_1.PDF]

Supplementary Material

PU.1 is required for the developmental progression of multipotent progenitors to common lymphoid progenitors

Swee Heng Milon Pang[1,2], Carolyn A de Graaf[1,2], Douglas J Hilton[1,2], Nicholas D Huntington[1,2], Sebastian Carotta[1,2,3*], Li Wu[1,2,4*] and Stephen L Nutt[1,2*]

[1] The Walter and Eliza Hall Institute of Medical Research, Parkville, VIC Australia. [2] Department of Medical Biology, University of Melbourne, Parkville, VIC Australia. [3] Boehringer Ingelheim RCV, Oncology Research, Vienna, Austria. [4] Institute for Immunology, Tsinghua University School of Medicine, Beijing, China.

* These authors contributed equally to this study.

Correspondence should be addressed to Stephen Nutt (nutt@wehi.edu.au) and Li Wu (wuli@tsinghua.edu.cn).

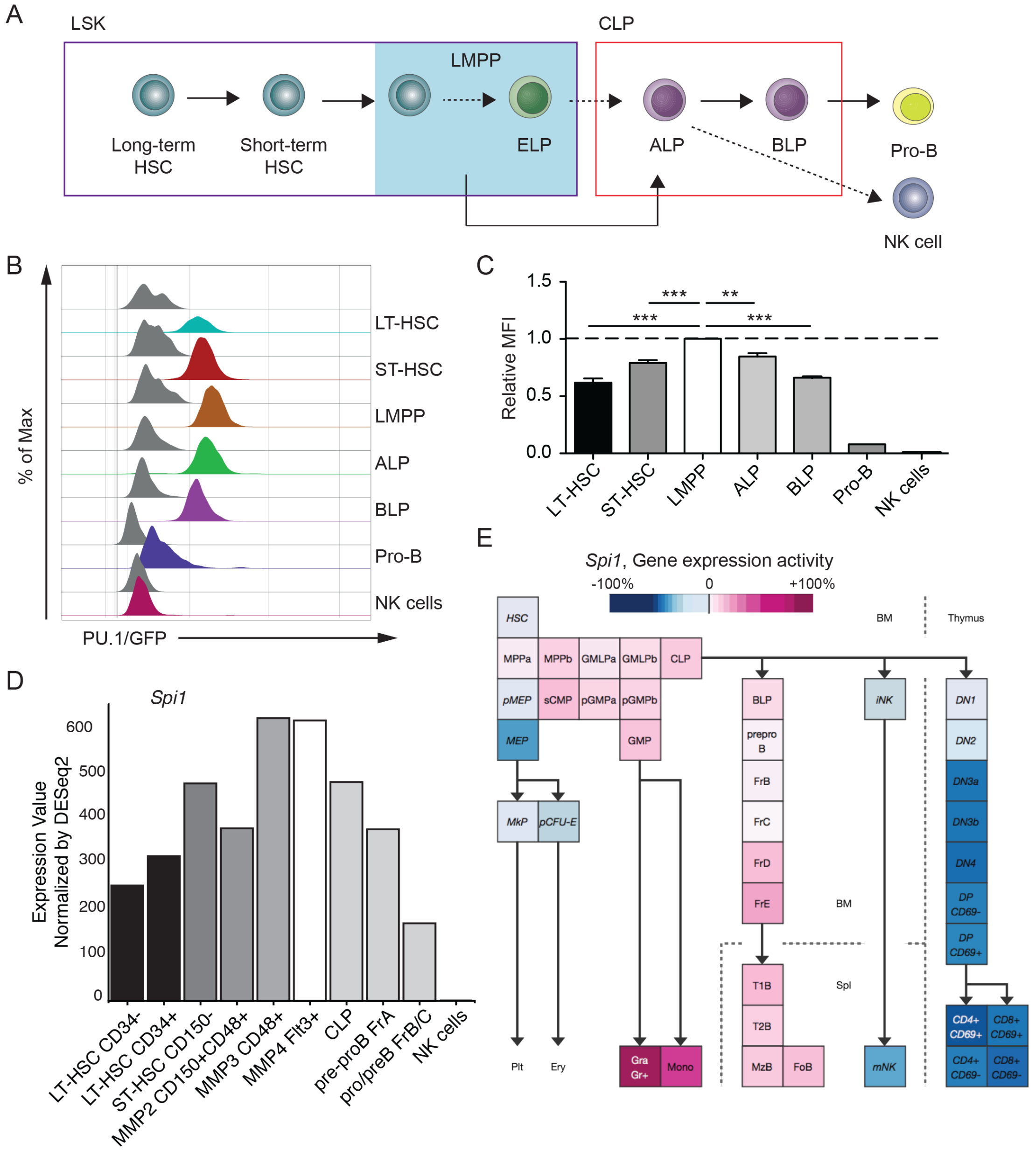

Figure S1. The regulation of PU.1 expression in lymphoid progenitors. **(A)** Schematic diagram of early lymphopoiesis described in this study. LT-HSC, long term-hematopoietic stem cell (Lin^{-}Sca1^{+}c-Kit^{high}CD34^{-}Flt3^{-}), ST-HSC, short term-hematopoietic stem cell (Lin^{-}Sca1^{+}c-Kit^{high}CD34^{+}Flt3^{-}), LMPP, lymphoid primed multipotent progenitor (Lin^{-}Sca1^{+}c-Kit^{high}CD34^{+}Flt3^{+}), ELP, early lymphoid progenitor (Lin^{-}Sca1^{+}c-Kit^{high}Rag1/GFP^{+}) ALP, all-lymphoid progenitor (Lin^{-}c-Kit^{int}IL-7R^{+}Flt3^{+}Ly6D^{-} or Lin^{-}c-Kit^{int}Rag1/GFP^{+}Ly6D^{-}), BLP, B-cell biased lymphoid progenitor (Lin^{-}c-Kit^{int}IL-7R^{+}Flt3^{+}Ly6D^{+} or Lin^{-}c-Kit^{int}Rag1/GFP^{+}Ly6D^{+}), Pro-B cells (CD19^{+}B220^{+}IgM^{-}c-Kit^{+}) and natural killer (NK) cells (NK1.1^{+}CD49b^{+}TCRβ^{-}). (B) Expression of PU.1/GFP in the indicated lineage-depleted (Lin^{-}) BM progenitor populations of *Spi1*$^{gfp/gfp}$. Pro-B cells (PU.1/GFP^{low}) and NK cells (PU.1/GFP^{-}) are shown for reference. Gray histograms show corresponding population from C57Bl/6 mice. **(C)** Quantitation of PU.1/GFP expression by each cell population shown in **(B)**. Data were normalized for relative mean fluorescence intensity (MFI) of LMPPs (set as 1). Graph depicts the MFI ± SD

from three experiments. p values compare the indicated groups using a paired *t*-test. ** p<0.01, *** p<0.001. **(D)** Normalized expression of *Spi1* mRNA determined by RNA sequencing from the indicated cell populations. Data is the mean of 2 biological replicates and is from *ImmGen* database. The population labelled MMP4 Flt3$^+$ (unshaded bar) corresponds to the LMPP in **(A-C)**. **(E)** Expression of *Spi1* mRNA from *Gene Expression Commons* database. Data shows the "gene expression activity" of the probe corresponding to *Spi1* in the indicated hematopoietic cell populations (using the Weissman laboratory model) relative to 11939 individual Affymetrix microarray datasets. The relative "gene expression activity" is shown color-coded in the hematopoietic cell populations. This data again shows *Spi1* expression being low in HSCs, increasing in MMP, granulocyte-macrophage progenitor (GMP) and CLP populations, before decreasing in B cell progenitors (pre-proB, FrB, FrC).

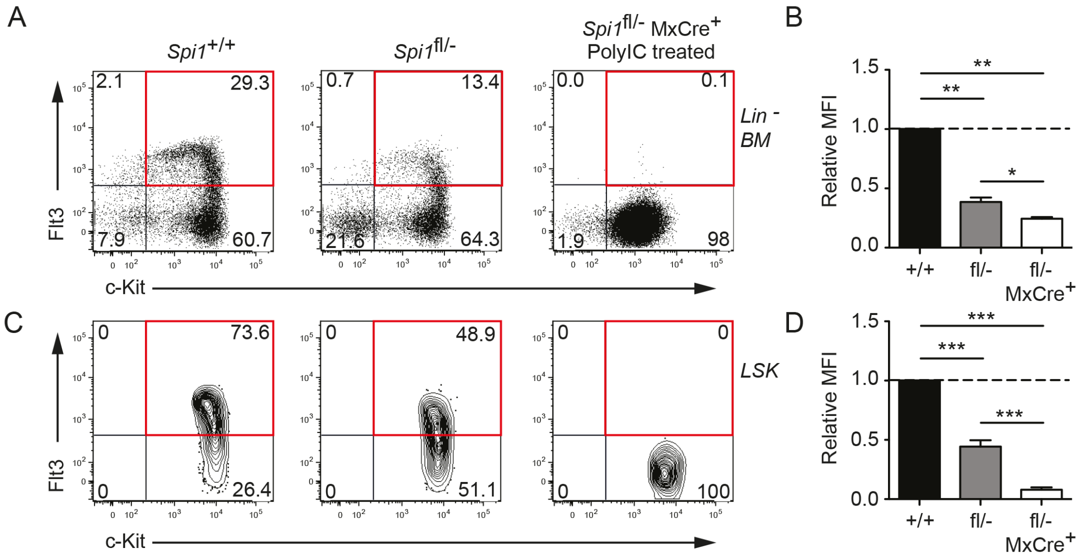

Figure S2. The regulation of Flt3 by PU.1. **(A-B)** Lin$^-$ BM and **(C-D)** LSK (Lin$^-$ Sca1$^+$c-Kit^{high}) cells from *Spi1* wild type (+/+), heterozygous (fl/-) and polyIC treated fl/- MxCre$^+$ mice were analyzed for Flt3. Numbers in **(A)** and **(C)** indicate the proportion of cells in each quadrant. Flt3 expression by **(B)** Lin$^-$ BM and **(D)** LSK cells of indicated genotypes were measured as the relative MFI ± SD (set as 1 for +/+ cells) from between 3-7 mice per genotype. p values compare the indicated groups using an unpaired *t-test*. * p<0.05, ** p<0.01, *** p<0.001.

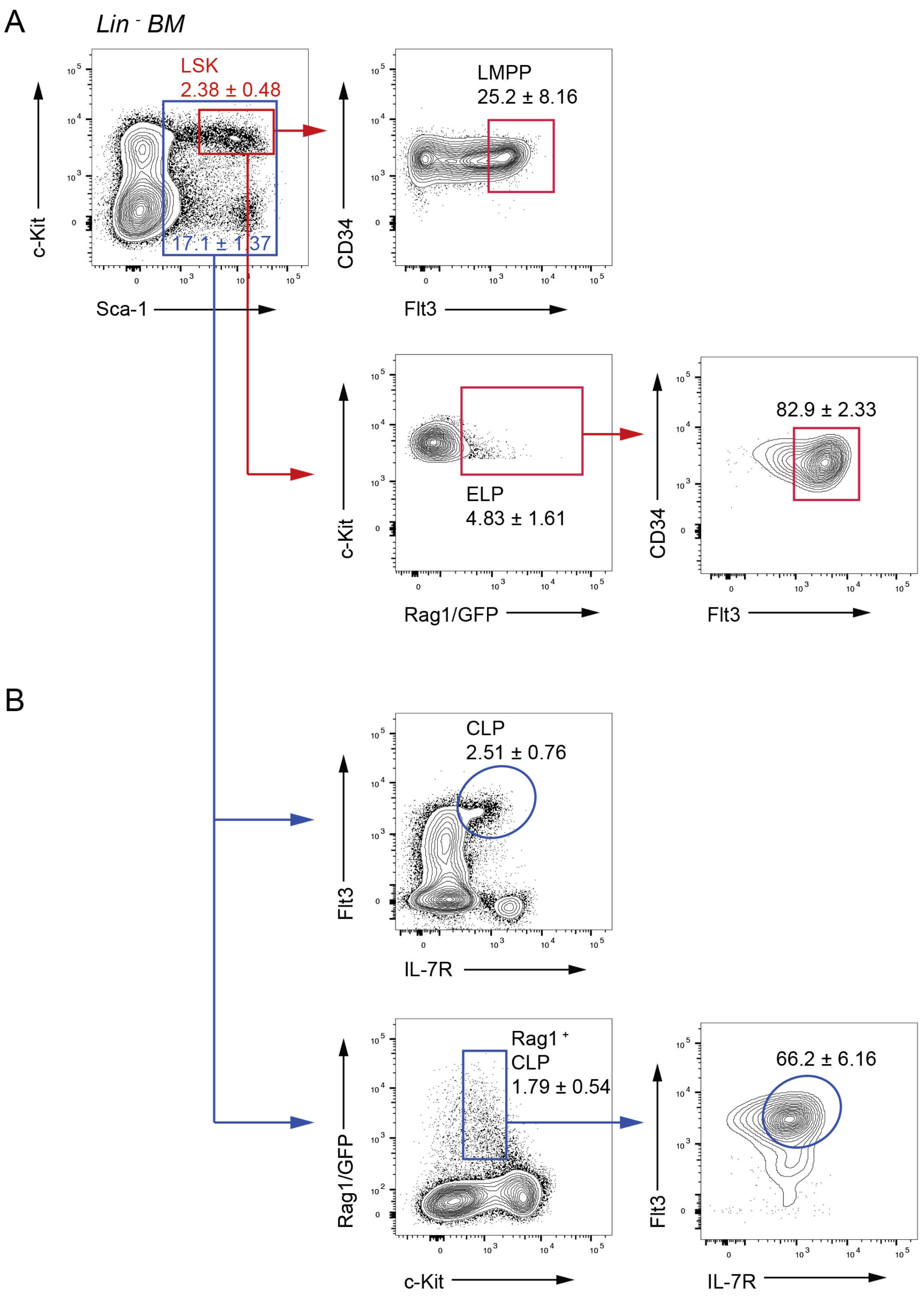

Figure S3. Use of Rag1/GFP to identify early lymphoid progenitors in the BM. Lineage-depleted (Lin^-) BM from *Rag1*$^{gfp/+}$ mice were isolated and analyzed to identify **(A)** Rag1/GFP$^+$ ELPs and **(B)** Rag1/GFP$^+$ CLPs. Comparison of gated ELPs and Rag1/GFP$^+$ CLPs to the established LMPP and CLP populations is shown. Numbers are mean proportion of gated cells ± SD from 3-5 mice.

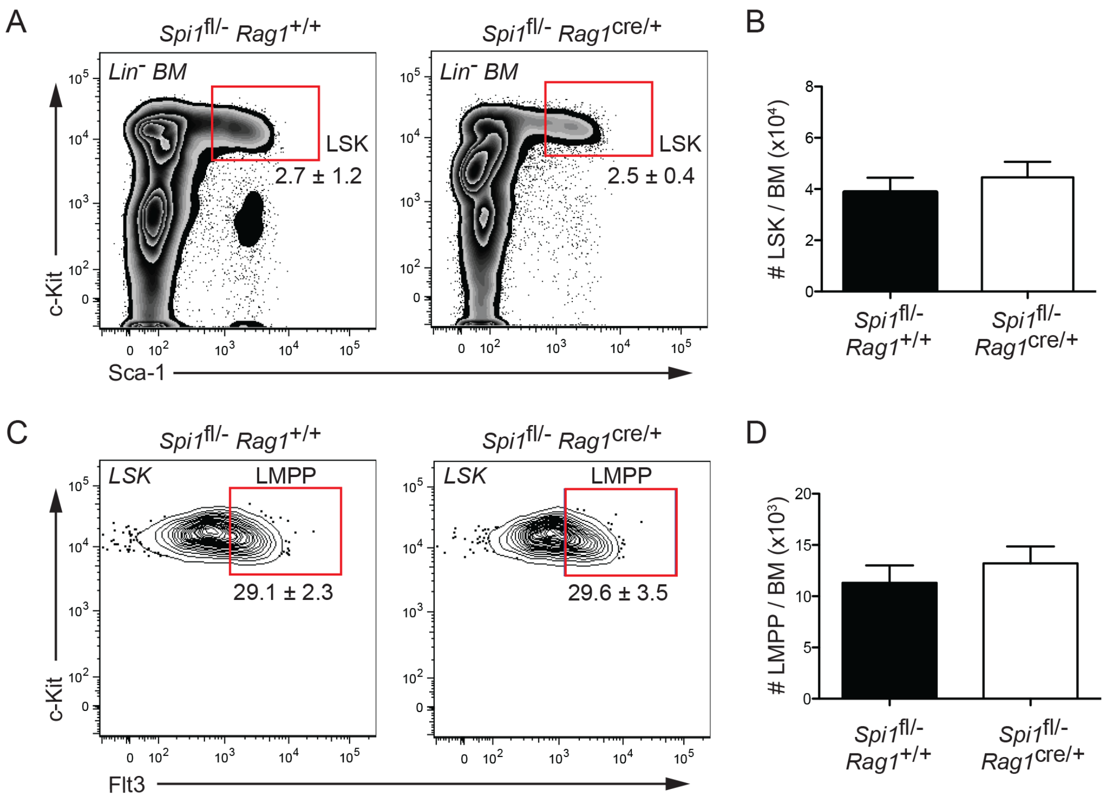

Figure S4. Normal frequency of LSK and LMPP populations in *Spi1*$^{fl/-}$*Rag1*$^{cre/+}$ mice. **(A-D)** Lin^{-} BM from *Spi1*$^{fl/-}$*Rag1*$^{+/+}$ and *Spi1*$^{fl/-}$*Rag1*$^{cre/+}$ mice were analyzed for the **(A)** frequency (in Lin^{-} BM) and **(B)** number of LSK cells and **(C)** frequency (within the LSK gate) and **(D)** number of LMPPs. Boxes show the position of gating for the cell type being analyzed. Data in are the mean ± SD from between 9-13 mice per genotype.

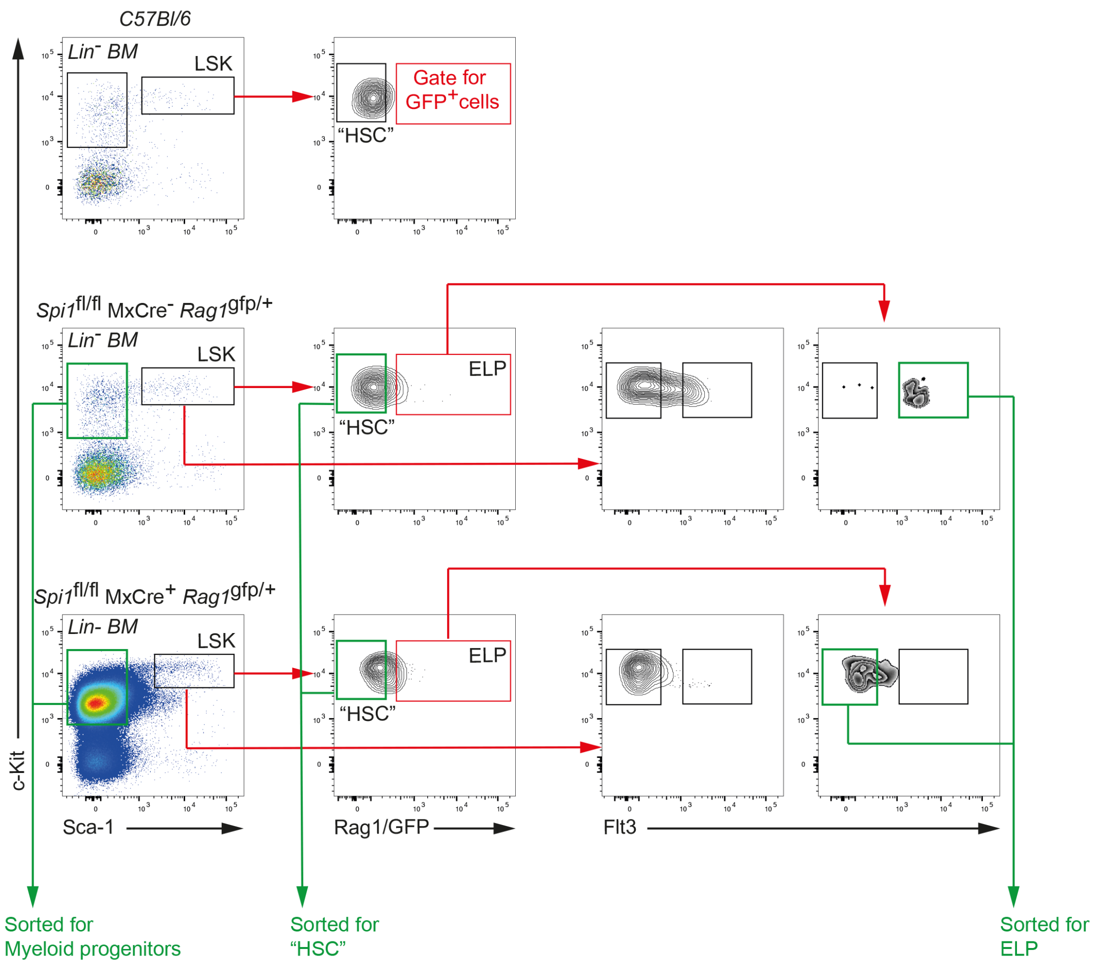

Figure S5. Sorting strategy for hematopoietic progenitors for gene expression analysis in Figure 5E. *Spi1*$^{fl/fl}$MxCre^{-}*Rag1*$^{gfp/+}$ and *Spi1*$^{fl/fl}$MxCre^{+}*Rag1*$^{gfp/+}$ mice were injected with polyIC on days 0 and 3, and analyzed by flow cytometry on day 14. Lin^{-} BM from C57Bl/6 mice was used as a control for GFP expression. Flt3 expression was included to confirm the deletion of PU.1 in the ELPs. "HSC", ELP and myeloid progenitors were isolated as indicated.

TABLE S1. Oligonucleotide primers used for quantitative real-time RT-PCR

Gene	Primer	Sequence (5'-3')
Csf1r	Forward	ATGAGTCCCTCTTCACTCCG
	Reverse	ACCTTCAGCACTGCATCTTC
Dntt	Forward	GAAGATGGGAACAACTCGAAGAG
	Reverse	CAGGTGCTGGAACATTCTGGGAG
Cd79a	Forward	GCCAGGGGGTCTAGAAGCCC
	Reverse	TCACTTGGCACCCAGTACAA
Oct2	Forward	CCCGGGCTGGGCTTCCTACACAG
	Reverse	GTAGCTGGTCGGCTTTCCCGGGCT
Pcp4l1	Forward	TCACGTATCTCATTCCTTCATCC
	Reverse	GCTCCTGGATTTAGGTCAGC
Pf4	Forward	CTCTTGACATGAGCGTCGCTGCGG
	Reverse	CTTGATCACCTCCAGGCAGGTGAA
Tek	Forward	TGTCAATCAGGCCTGGAAATAC
	Reverse	GAGGAGGGAGAATGTCACTAAGG
Slc22a18	Forward	GCTTCGGCGTCGGAGTCAT
	Reverse	AGCCTGGGCGTCAGTTTT
Aqp1	Forward	CTGCTGGCGATTGACTACACTG
	Reverse	GGTTTGAGAAGTTGCGGGTGAG
Slpi	Forward	CACATATACCCTCACAGCAC
	Reverse	CTATCAAAATCGGAGCCTGC
Nmes1	Forward	TCTACACTAAAGTCATCATGGGCGTTTT
	Reverse	AGAGGAGTGGTGAGCAATCATCTGG
Hprt	Forward	GGGGGCTATAAGTTCTTTGC
	Reverse	TCCAACACTTCGAGAGGTCC
